# *MEIS1* down-regulation by MYC mediates prostate cancer development through elevated *HOXB13* expression and AR activity

**DOI:** 10.1038/s41388-020-01389-7

**Published:** 2020-07-17

**Authors:** Nichelle C. Whitlock, Shana Y. Trostel, Scott Wilkinson, Nicholas T. Terrigino, S. Thomas Hennigan, Ross Lake, Nicole V. Carrabba, Rayann Atway, Elizabeth D. Walton, Berkley E. Gryder, Brian J. Capaldo, Huihui Ye, Adam G. Sowalsky

**Affiliations:** 1 Laboratory of Genitourinary Cancer Pathogenesis, National Cancer Institute, NIH, Bethesda, MD 20892 USA; 2 Genetics Branch, National Cancer Institute, NIH, Bethesda, MD 20892 USA; 3 Department of Pathology, Beth Israel Deaconess Medical Center, Boston, MA 02215 USA; 4 Present Address: Department of Pathology, University of California Los Angeles, Los Angeles, CA 90095 USA

**Keywords:** Cancer genetics, Prostate cancer

## Abstract

Localized prostate cancer develops very slowly in most men, with the androgen receptor (AR) and MYC transcription factors amongst the most well-characterized drivers of prostate tumorigenesis. Canonically, MYC up-regulation in luminal prostate cancer cells functions to oppose the terminally differentiating effects of AR. However, the effects of MYC up-regulation are pleiotropic and inconsistent with a poorly proliferative phenotype. Here we show that increased MYC expression and activity are associated with the down-regulation of *MEIS1*, a HOX-family transcription factor. Using RNA-seq to profile a series of human prostate cancer specimens laser capture microdissected on the basis of MYC immunohistochemistry, MYC activity, and *MEIS1* expression were inversely correlated. Knockdown of *MYC* expression in prostate cancer cells increased the expression of *MEIS1* and increased the occupancy of MYC at the *MEIS1* locus. Finally, we show in laser capture microdissected human prostate cancer samples and the prostate TCGA cohort that *MEIS1* expression is inversely proportional to AR activity as well as *HOXB13*, a known interacting protein of both AR and MEIS1. Collectively, our data demonstrate that elevated MYC in a subset of primary prostate cancers functions in a negative role in regulating *MEIS1* expression, and that this down-regulation may contribute to MYC-driven development and progression.

## Introduction

Locally advanced prostate cancers harbor a limited number of recurrently altered genes whose expression change at the earliest stages of tumor development. These include down-regulation of the tumor suppressors *NKX3-1* and *PTEN* (often due to genomic deletion), up-regulation of *ERG* (due to fusion with *TMPRSS2*), and up-regulation of *MYC*, which often co-occurs with a single-copy gain of chromosome 8q24 [1–5]. Although up-regulation of *MYC* in most neoplastic tissues is a very early event that contributes to self-renewal and proliferation, localized prostate cancer (PCa) is not proportionally as hyperproliferative as metastatic prostate cancers which frequently harbor focal amplification of *MYC* [6, 7]. In part, the effects of the androgen receptor (AR) in terminally-differentiated luminal prostate cells are disrupted by MYC and other co-factors including FOXA1 and HOXB13 to re-engage proliferative processes during tumorigenesis [7–11].

Recently, increased awareness that the vast majority of prostate cancers are indolent has led to increased molecular profiling of tumor biopsies prior to definitive treatment. Although MYC expression has been observed in the cancer precursor high-grade prostatic intraepithelial neoplasia, MYC expression in indolent-appearing tumor cells predicts the presence of higher-grade disease and is associated with poor differentiation [3–5]. In localized prostate cancers, up-regulated MYC has been further associated with alterations in nucleoli structure, concomitant with increased biogenesis of ribosomal RNA, increased purine metabolism, and expression of the telomere RNA subunit *TERC* [12–14]. These effects contrast sharply with the phenotype of highly amplified MYC, which is enriched in metastatic and treatment-resistant prostate cancers, and mirrors the role of MYC in other cancer types via effects on AKT to contribute toward cell division and survival [15–17]. Nonetheless, even in primary PCa, MYC protein expression is diffuse and heterogeneous [3].

We sought to examine the genetic contribution of MYC to the development and progression of primary PCa in the context of dysregulated growth by using anti-MYC immunohistochemistry (IHC) and performing laser capture microdissection on populations of human prostate tumor cells with varying expression of MYC protein. We show using transcriptome profiling that increased MYC activity is inversely correlated with expression of Myeloid Ecotropic viral Insertion Site 1 (*MEIS1*), a transcription factor that interacts with and regulates the activity of HOX homeodomain transcription factors, including HOXB13 [18–20]. We determined that MYC binding to the *MEIS1* locus decreases as MYC levels increase, and that *MEIS1* expression is negatively correlated with *HOXB13* expression and AR activity.

## Results

### MYC activity is weakly associated with proliferation in primary prostate cancer

**Fig. 1:**
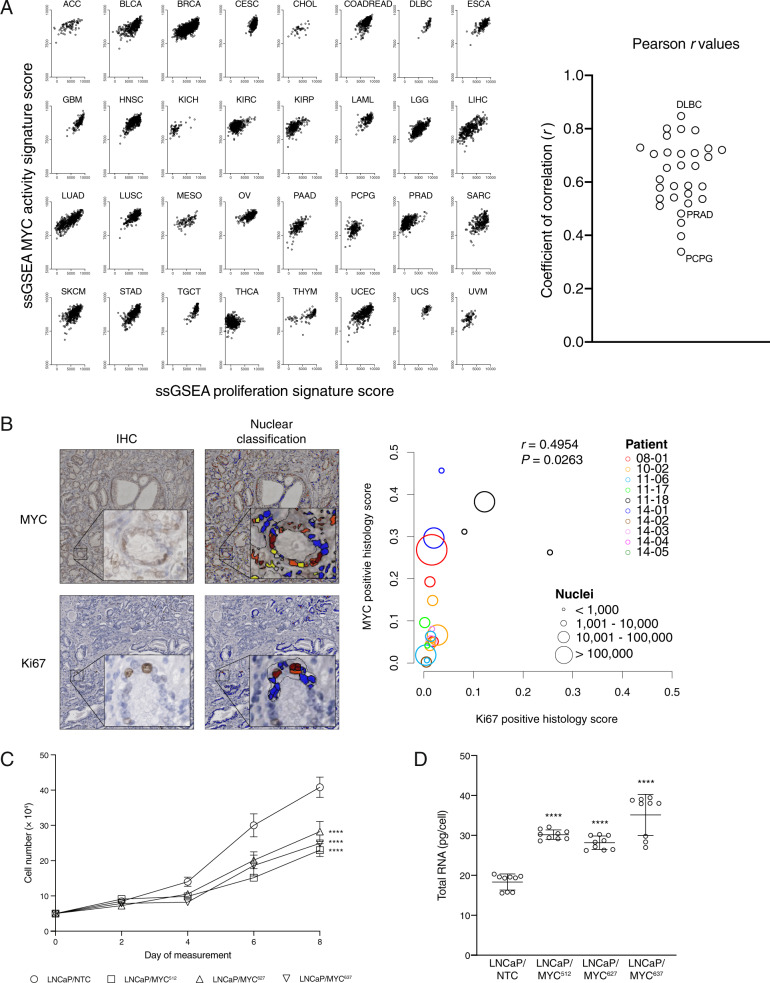
MYC and proliferation in human cancers. **a** Left: correlation of the 54-gene ssGSEA MYC activity signature score and the 17-gene ssGSEA proliferation signature score for each tumor type from the TCGA pan-cancer cohort. All scatter plots are on the same scale. Right: plot of Pearson *r* coefficients of correlation from each scatter plot shown on the left. All tumor types showed a positive correlation, ranging from 0.3386 (PCPG: pheochromocytoma and paraganglioma) to 0.8473 (DLBC: diffuse large B-cell lymphoma). PRAD: prostate adenocarcinoma, *r* = 0.4822. All correlations were significant at *P* < 0.001 except THCA (thyroid carcinoma; *P* = 0.5543). **b** Left: representative IHC staining used as input for automated image analysis with Definiens Tissue Studio and the processed file used for quantification of histologic score via nuclear classification, for anti-MYC and anti-Ki67 immunostains. Right: unweighted scatter plot and Pearson correlation of the quantified intensities of anti-Ki67 vs. anti-MYC stain. Each open circle represents a contiguous quantified region, color-coded by patient. Multiple regions from the same patient indicate multiple noncontiguous regions were measured, and the size of each circle indicates the number of nuclei considered for the analysis. **c** Assessment of cell growth in vitro as a function of *MYC* knockdown. LNCaP cells expressing non-targeting or MYC-targeting hairpins were seeded at a density of 50,000 cells per well and counted every 2 days in triplicate using a hemocytometer by three different individuals blinded to the identities of the cultures. Data shown represents the mean ± standard error of ten independent experiments. ****P < 0.0001 for each cell line vs. control by two-way ANOVA. **d** RNA content as a function of *MYC* knockdown. Total RNA was extracted from 1 × 10^6^ pelleted LNCaP cells expressing non-targeting or *MYC*-targeting hairpins. RNA concentrations were measured by Nanodrop and total RNA was calculated per cell. Data represents mean ± standard deviation of three independent experiments conducted in triplicate, with open circles representing individual replicate RNA quantity values (****P < 0.0001 for each cell line vs. control by two-way ANOVA).

Although MYC orchestrates a broad range of biological functions, it has been shown in many cancers that MYC drives tumorigenesis by potentiating or stimulating cell growth and proliferation [21]. To assess the relationship between MYC activity and proliferation across cancer types, we evaluated MYC activity and cell proliferation rate in TCGA pan-cancer cohort using ssGSEA with MYC activity and proliferation signatures [22]. As shown in Fig. 1a, signatures for MYC activity and proliferation were positively correlated in all cancers with the vast majority showing strong positive correlations (*r* > 0.5). However, prostate adenocarcinoma (PRAD) was below this cut-off as one of the weakest correlating cancer types (*r* = 0.4822, 95% C.I. 0.4112–0.5472, *P* < 0.0001). Repeating this analysis with a prostate cancer-specific gene set generated by MYC overexpression in LNCaP cells [11], PRAD correlation with proliferation increased modestly (*r* = 0.5609, 95% C.I. 0.4972–0.6186, *P* < 0.0001), but was still below the median correlation coefficient (Supplementary Fig. 1). To validate this observation, we screened a new cohort of treatment naïve Gleason score 7 prostate tumors for MYC and Ki67 expression by IHC. Although MYC was expressed in a majority of tumor cells, Ki67 expression was limited to <5% of cells (Fig. 1b, left), consistent with median Ki67 indices from large cohorts of GS7 tumors in the 2–4% range [23, 24]. We then applied automated image analysis to quantify millions of nuclei across 20 tumor foci from ten different cases. While the MYC positive histology score ranged from 0 (low expression) to 0.46 (high expression), the Ki67 positive histology score was <0.1 in 18 out of 20 foci (Fig. 1b, right). An unweighted Pearson correlation demonstrated that MYC IHC was weakly but positively correlated with Ki67 (*r* = 0.4954, 95% C.I. 0.06772–0.7693, *P* = 0.0263), similar to what we observed in the PRAD TCGA cohort, indicating that the transcriptional signatures applied to the larger dataset accurately recapitulated MYC and Ki67 protein abundance. Because high Ki67 expression was not associated with high MYC expression, proliferation may have been dependent upon MYC activity, but MYC expression alone was not sufficient for proliferation.

To further examine the role of MYC in prostate tumorigenesis, we knocked down MYC in LNCaP cells, which had physiologically-elevated levels of MYC. Like primary PCa, LNCaP cells express AR, are androgen sensitive, and grow slowly despite elevated MYC [25]. Using MYC-knockdown cells as a model of MYC-low PCa cells, we show in Supplementary Fig. 2 that we achieved ~50% reduction in *MYC* transcript and MYC protein expression. As anticipated, knockdown of *MYC* was associated with a modest decrease in cell number at each time point (Fig. 1c) but the decrease in cell proliferation was not proportional to the decrease in *MYC* expression. The relationship between MYC and proliferation in other cancer types also stipulates a role for MYC as a universal amplifier of transcription [26], alleviating constraints on cell growth and proliferation [27]. However, we observed the opposite: the total amount of cellular RNA increased when *MYC* was knocked down in LNCaP cells (Fig. 1d). Together, these findings indicate that the cellular behavior of MYC in PCa contrasts with other tumor types, and that MYC does not act solely in a proliferative capacity.

### Increased MYC is associated with specific up- and down-regulation of target genes

**Fig. 2:**
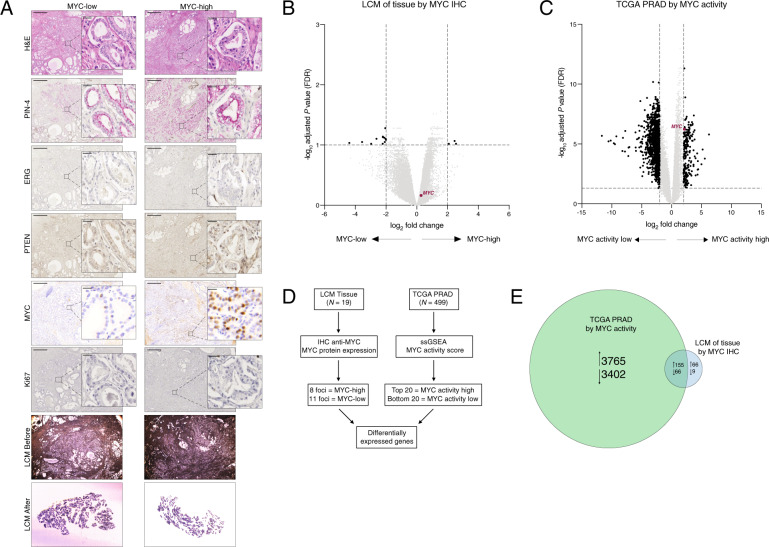
Effect of MYC levels on gene expression. **a** Representative IHC of case 14-03 showing heterogeneity of MYC expression within a single patient. Serial sections were stained with H&E and PIN-4 cocktail to distinguish benign glands and high grade prostatic intraepithelial neoplasia from tumor foci; anti-ERG and anti-PTEN immunostains were performed to select regions with concordant phenotypes. Micrographs of stained tissue before and after laser capture microdissection (LCM) are also shown. Scale bar: 500 μm; inset scale bar: 20 μm. **b** Volcano plot depicting differentially expressed genes in MYC-high vs. MYC-low tumors in the LCM cohort, with the false discovery rate (adjusted −log_10_ *P* value) on the *y*-axis and the log_2_-transformed fold-change (MYC-high vs. MYC-low) on the *x*-axis. Expression of *MYC* is shown in red. Black dots represent genes with fold-change ≥ ±4 and adjusted *P* value < 0.10 for Welch’s *t* test. **c** Volcano plot depicting differentially expressed genes in MYC activity high vs. MYC activity low tumors in the TCGA PRAD cohort, with the false discovery rate (adjusted −log_10_ *P* value) on the *y*-axis and the log_2_-transformed fold-change (MYC activity high vs. MYC activity low) on the *x*-axis. Expression of *MYC* is shown in red. Black dots represent genes with fold-change ≥ ±4 and adjusted *P* value < 0.05 for Welch’s *t* test. **d** Flowchart of the strategy used to identify lists of differentially expressed genes from the LCM and TCGA PRAD cohorts. **e** Euler diagram depicting distinct and shared differentially expressed genes between the LCM and TCGA PRAD cohort. Circles are drawn to scale.

We hypothesized that analysis of tumors with different levels of MYC expression would identify genes that may contribute to MYC activity in PCa pathogenesis. Using samples of human radical prostatectomy specimens stained with anti-MYC, we identified concomitant regions of high-and low-MYC expression in the same patients. We subjected these within-patient sets of tumor foci to laser capture microdissection (Fig. 2a), controlling for PTEN and ERG status by confirming their concordance within each case. Of the 19 foci microdissected, we designated 42% MYC-high (*n* = 8) and 58% MYC-low (*n* = 11). We performed RNA-seq on these cases and derived a limited gene set of 293 up- or down-regulated genes, with only 15 genes showing expression changes of fourfold or more with adjusted *P* values < 0.10 (Fig. 2b and Supplementary Table 2). Consistent with our observation of increased RNA in LNCaP cells expressing MYC knockdown hairpins (see Fig. 1d), we observed more genes down-regulated than up-regulated in MYC-high vs. MYC-low tumor foci.

To further refine genes of potential interest, we analyzed a second dataset comprised of whole transcriptome sequencing from 499 primary tumors in the TCGA PRAD cohort. Using the mRNA expression values of genes in the MYC activity signature (see Fig. 1a and Supplementary Table 1), we compared the top 20 and bottom 20 ranked cases based on average median absolute deviation-modified *z*-score (Supplementary Fig. 3), and established a second list of differentially expressed genes (Fig. 2c and Supplementary Table 3). As these cases were compared between patients rather than within-patient, a far greater number of genes were differentially expressed (1346) with fold-change of at least 4 and adjusted *P* values < 0.05.

When comparing differentially expressed genes between MYC-high vs. MYC-low tumors, *MYC* expression was consistently greater in the MYC-high group in the LCM primary PCa cohort (Fig. 2b and Supplementary Table 2). As MYC RNA and protein expression often do not correlate due to the tight posttranslational regulation of MYC [28, 29], the statistically insignificant increase of *MYC* expression in the MYC-high group was not unexpected. However, *MYC* was amongst the up-regulated genes in the MYC activity high group, confirming proper stratification in the TCGA PRAD cohort as well (Fig. 2c and Supplementary Table 3). Taking an inclusive approach to combine both datasets, we identified 293 and 7390 differentially expressed genes between MYC-high and MYC-low prostate tumors in the LCM tissue and TCGA PRAD cohorts, respectively (Fig. 2d, e) that were concordantly regulated in the same direction. Of these, 221 were in common at a false discovery rate threshold of 0.10 for LCM tissue and 0.05 for TCGA PRAD (Fig. 2e and Supplementary Table 4).

### *MEIS1* is negatively associated with *MYC* expression and MYC activity

**Fig. 3:**
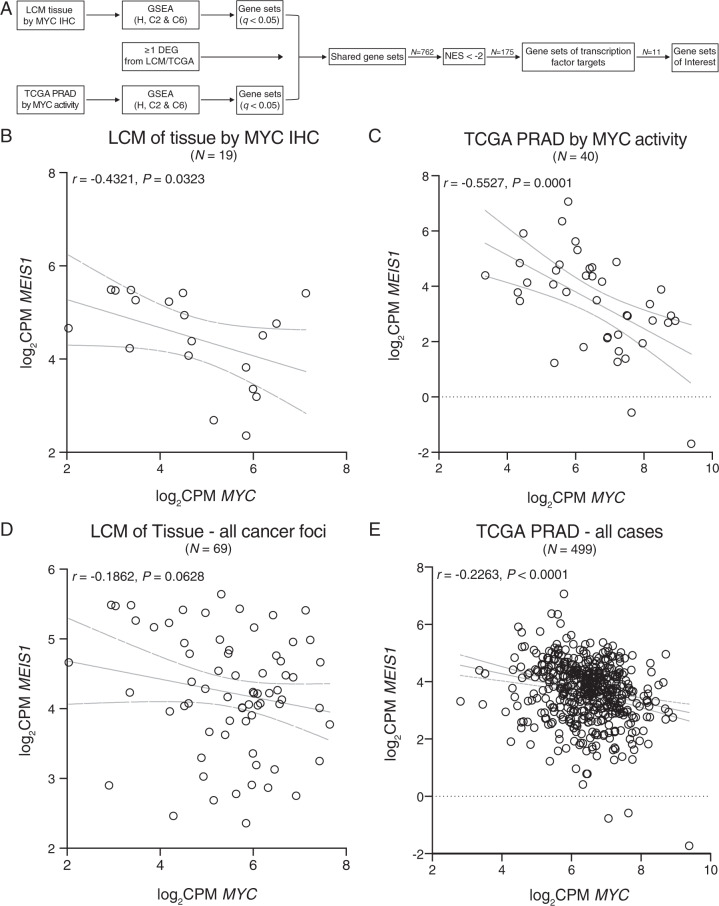
Association between *MYC* and *MEIS1* expression. **a** Flowchart of the strategy used to identify potential MYC target pathways based on differentially expressed genes. **b**–**e** Correlation of the log_2_ CPM expression levels between *MYC* and *MEIS1* in the initial LCM cohort (*N* = 19, **b**), the initial TCGA PRAD cohort (*N* = 40, **c**), the entire LCM primary PCa cohort (*N* = 69, **d**), and the entire TCGA PRAD cohort (*N* = 499, **e**). The gray dotted lines represent the 95% confidence intervals for each correlation.

Given that we observed more genes down-regulated in MYC-high tumors than we would have expected if MYC were functioning as a genome-wide transcriptional amplifier, we hypothesized that collective analyses of gene expression might reveal coordinated down-regulation of biological processes that may contribute to MYC-driven PCa tumorigenesis. We therefore performed gene set enrichment analysis (GSEA) using both the LCM and TCGA cohort datasets as inputs, limiting our analyses to gene sets with differentially expressed genes shared by the LCM and TCGA cohorts. As depicted in Fig. 3a, our initial comparative analyses demonstrated significant and concordant enrichment of 762 gene sets (Supplementary Table 5). To narrow our focus further, we refined our search only to include gene sets based on ChIP-seq target discovery studies with the premise that increased MYC activity would misregulate a multitude of transcriptionally-driven pathways. These analyses identified 11 gene sets, a preponderance of which were associated with development and survival (Table 1).

**Table 1:** List of common gene sets negatively enriched in MYC-activity high vs. low comparing LCM tissue and TCGA cohorts.

	LCM tissue		TCGA	
MSigDB gene set	*NES*	*Q* value	*NES*	*Q* value
Onder CDH1 Targets 3 DN	−2.041	4.27E-03	−2.193	6.96E-03
Ono AML1 Targets DN	−2.154	2.18E-03	−2.135	6.96E-03
Klein Targets of BCR ABL1 Fusion	−2.192	1.80E-03	−2.274	6.96E-03
Hess Targets of HOXA9 and MEIS1 DN	−2.206	1.63E-03	−2.192	6.96E-03
Takeda Targets of NUP98 HOXA9 Fusion DN	−2.216	1.43E-03	−2.005	6.96E-03
Phong TNF Targets UP	−2.222	1.41E-03	−2.207	6.96E-03
Kim GLIS2 Targets UP	−2.431	1.75E-04	−2.066	6.96E-03
Hecker IFNB1 Targets	−2.497	1.50E-04	−2.086	6.96E-03
Servitja Islet HNF1A Targets UP	−2.542	8.51E-05	−2.164	6.96E-03
Vilmas NOTCH1 Targets UP	−2.575	8.93E-05	−2.342	6.96E-03
Gaurnier PSMD4 Targets	−2.768	0.00E + 00	−2.311	6.96E-03

*NES* normalized enrichment score.

Of note, gene sets related to NOTCH, RUNX1, HOXA9, and TNF were negatively enriched in MYC-high vs. MYC-low tumors. This suggested that one or more transcription factors exert effects in opposition to MYC, and may be guiding MYC-mediated misregulation of these pathways. In seeking a gene or common regulator between these gene sets, we identified the transcription factor MEIS1 as an important regulator in each of the aforementioned pathways. For example, MEIS1 has been shown to regulate genes in the NOTCH pathway [30] and sensitizes cells to TNF [31]. Moreover, MEIS1 is essential for the expression of genes driven by the HOXA9-NUP98 fusion in acute myeloid leukemia [32–34]. Therefore, we examined whether *MEIS1* expression was associated with increased MYC activity as a proxy for its role in PCa development.

We observed that *MEIS1* expression was reduced in MYC-high cases compared with MYC-low tumors, for both the LCM and TCGA cohorts (Supplementary Tables 2 and 3, respectively). At a case-by-case level, *MEIS1* expression was negatively correlated with *MYC* (Fig. 3b; *r* = −0.4321, 95% C.I. −0.7409–0.0275, *P* = 0.0323) and MYC activity (Fig. 3c; *r* = −0.5527, 95% C.I. −0.7372 to −0.2713, *P* = 0.0001) in the LCM and TCGA cohorts, respectively. To assess whether this association occurred in unselected populations of primary PCa, we analyzed RNA-seq data from an additional 69 microdissected tumor foci and the entire TCGA PRAD cohort (*n* = 499). Although weaker than the MYC-selected cohorts, the negative relationship between *MEIS* and *MYC* expression was still observed (Fig. 3d, e).

### MYC negatively regulates *MEIS1* expression

Recently, Bhanvadia et al., postulated that higher *MEIS1* expression conferred a less aggressive PCa phenotype [19]. Based on these results, we hypothesized that repression of *MEIS1* expression by MYC may contribute to the aggressivity of MYC-driven PCa. We engineered LNCaP cells with a non-targeting hairpin as MYC-high and LNCaP cells with three different *MYC*-targeting hairpins as MYC-low (see Supplementary Fig. 2), with variable efficiency of *MYC* knockdown to replicate the biological variability observed in patient samples. We then performed ChIP-seq against MYC in these cells to generate genome-wide site maps and ascertain chromatin occupancy at *MEIS1*. In these cells, we had observed increased overall transcriptional output (see Fig. 1d), and we controlled for this phenomenon using *Drosophila* chromatin spike-in controls.

The quality of our MYC ChIP DNA was assessed using Active Motif’s ChIP-IT qPCR Analysis Kit and primers targeting a MYC super-enhancer region prior to sequencing. As shown in Supplementary Fig. 4A, an enrichment of six- to eight-fold was achieved for each experiment and thus submitted for subsequent ChIP-seq analysis. We identified ~18,000 and 27,000 high confidence peaks (*P* < 1 × 10^−9^) in LNCaP/NTC and LNCaP/shMYC consensus sites (that is, sites common in all three *MYC* knockdown lines), respectively; and of these, 98% of all peaks were shared (Supplementary Fig. 4B). These sites overlapped with those reported in multiple publicly-available MYC ChIP-seq datasets ([11, 35–37]), with the extent of overlap ranging from 22 to 90% (Supplementary Table 6). Of note, when compared with another LNCaP MYC ChIP-seq, the overlap ranged from 77 to 90%, validating the technical results of our experiment. Applying functional annotation of binding sites, the majority of MYC-bound peaks were either promoter proximal or enriched for intronic/intergenic DNA in both control and *MYC* knockdown cell lines (Supplementary Fig. 4C). This distribution was also similar to that of previous MYC ChIP-seq datasets, providing orthogonal validation of our data (Supplementary Fig. 4D).

**Fig. 4:**
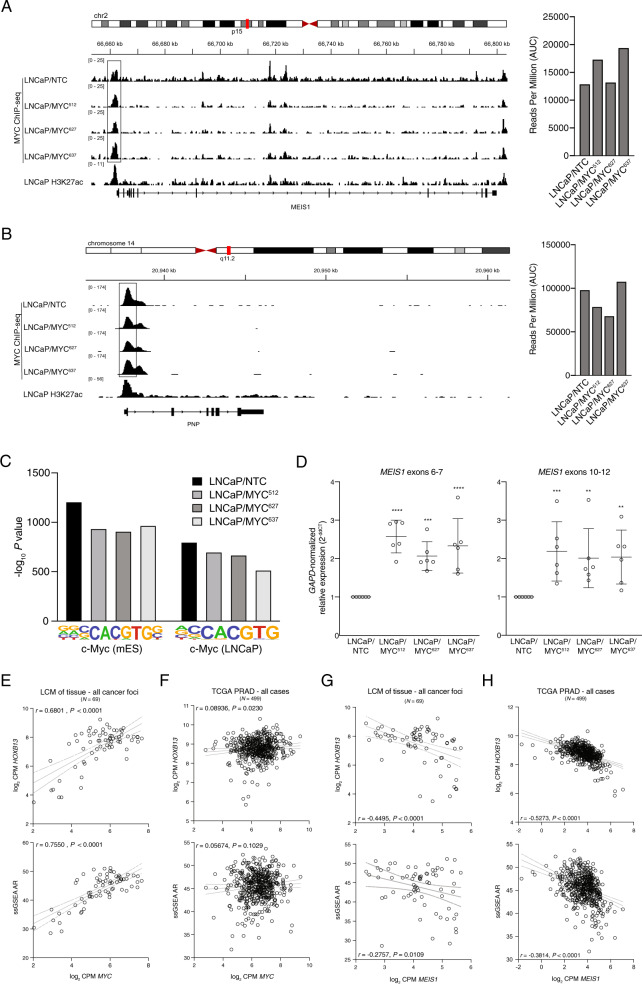
Decreased *MYC* expression increased MYC occupancy at *MEIS1* and *MEIS1* expression. **a** IGV depiction of MYC and H3K27ac binding events in LNCaP *MYC* knockdown cell lines, showing increased MYC occupancy at the *MEIS1* locus in cells harboring *MYC* knockdown. **b** IGV depiction of MYC and H3K27ac binding events in LNCaP *MYC* knockdown cell lines, showing decreased MYC occupancy at the *PNP* locus in cells harboring *MYC* knockdown. Graphs to the right of (**a**, **b**) depict the area under the curve (AUC) of read counts for the peaks outlined by the boxes. **c** The confidence of identifying known MYC binding motifs as identified by HOMER are shown for the LNCaP *MYC* knockdown cell lines, measured by −log_10_ *P* value for enrichment. *Q* values (adjusting for false discovery) for these enrichments are zero. **d** Quantitative reverse-transcription PCR of *GAPD*-normalized *MEIS1* transcript in LNCaP MYC knockdown cell lines relative to control, measuring across the splice boundary of exons 6–7 (left) or exons 10–12 (right). Bars and whiskers represent the mean ± standard deviation of six independent experiments conducted in triplicate, plotted individually as open circles (**P < 0.01; ***P < 0.001; ****P < 0.0001 by Student’s *t* test). **e**, **f** Correlation of the log_2_ CPM expression level for *MYC* with the log_2_ CPM expression level for *HOXB13* (top) and the 266-gene ssGSEA AR activity signature score (bottom) in the entire LCM cohort (*N* = 69, **e**) and the entire TCGA PRAD cohort (*N* = 499, **f**). The gray dotted lines represent the 95% confidence intervals for each correlation. **g**, **h** Correlation of the log_2_ CPM expression level for *MEIS1* with the log_2_ CPM expression level for *HOXB13* (top) and the 266-gene ssGSEA AR activity signature score (bottom) in the entire LCM cohort (*N* = 69, **g**) and the entire TCGA PRAD cohort (*N* = 499, **h**). The gray dotted lines represent the 95% confidence intervals for each correlation.

When we examined MYC ChIP-seq peaks in the vicinity of *MEIS1*, we observed that proportional MYC recruitment to *MEIS1* was increased 1.29-fold on average, relative to global binding in each of the three *MYC* shRNA lines relative to control (Fig. 4a). In contrast to *MEIS1*, we would anticipate that genes positively regulated by MYC would demonstrate reduced recruitment of MYC at their loci when MYC levels are reduced by shRNA, counter to what we observed in Fig. 4a. When we examined binding events at the known MYC-regulated gene *PNP*, we observed 1.17-fold less MYC recruitment, on average, to the *PNP* locus (Fig. 4b). For both *MEIS1* and *PNP*, ChIP-seq for H3K27ac marks in parental LNCaP cells also identified a peak at the same genomic position, indicating that the MYC ChIP-seq was identifying chromatin of active enhancer elements. Similar results were observed for two additional MYC targets, *NOLC1* and *EIF5A* (Supplementary Fig. 5A-B) of 1.28-fold less binding at each locus. The increased occupancy of MYC at *MEIS1* was far more pronounced when peaks were normalized to *Drosophila* spike-in controls (Supplementary Fig. 5C), although this also resulted in minimal differences between control and *MYC* knockdown for MYC binding at *PNP* (Supplementary Fig. 5D). Nonetheless, motif enrichment analysis at MYC-ChIP peaks showed decreases at canonical MYC binding sites upon *MYC* knockdown (Fig. 4c).

We then sought to determine if the observed increase in chromatin occupancy translated to altered *MEIS1* transcription by qRT-PCR. Knockdown of *MYC* resulted in increased abundance of the *MEIS1* transcript, consistent with the observed increase in MYC recruitment at *MEIS1* (Fig. 4d). Together, these results support the role of MYC in the negative regulation of *MEIS1* in primary PCa.

In PCa, MEIS1 functions to direct transcriptional specificity and activity of HOXB13 and act as a negative regulator of AR [20, 38]. Therefore, we next determined whether *HOXB13* expression or AR activity were altered in the context of MYC activity. Indeed, there were statistically significant positive correlations between *MYC* and *HOXB13* mRNA levels in both the entire LCM tissue cohort (Fig. 4e, top; *r* = 0.6801, 95% C.I. 0.5285–0.7897, *P* < 0.0001) and the entire TCGA PRAD cohort (Fig. 4f, top; *r* = 0.0894, 95% C.I. 0.0016–0.1758, *P* = 0.0230) although the strength of correlation in the TCGA cases was notably less than the purer LCM tissue cohort. We also observed a positive correlation between *MYC* mRNA levels and the ssGSEA scores for AR activity [39] in both the LCM cohort (Fig. 4e, bottom; *r* = 0.7550, 95% C.I. 0.6311–0.8413, *P* < 0.0001) and the TCGA cohort (Fig. 4f, bottom; *r* = 0.0567, 95% C.I. −0.0312–0.1438, *P* = 0.1029), although statistical significance was reached solely in the LCM cohort. Moreover, there was a statistically significant negative correlation between *MEIS1* and *HOXB13* expression in both the LCM cohort (Fig. 4g, top; *r* = −0.4495, 95% C.I. −0.6202 to −0.2381, *P* < 0.0001) and the TCGA cohort (Fig. 4h, top; *r* = −0.5273, 95% C.I. −0.5879 to −0.4608, *P* < 0.0001). This inverse association was also observed between *MEIS1* expression and AR activity in the LCM (Fig. 4g, bottom; *r* = −0.2757, 95% C.I. −0.4810–0.0417, *P* = 0.0109) and TCGA (Fig. 4h, bottom; *r* = −0.3814, 95% C.I. −0.4540 to −0.3038, *P* < 0.0001) cohorts. Given the potential impact of the *HOXB13* G84E SNP (rs138213197) on MEIS1 interaction, expression, and function [20], we performed SNV analysis of germline samples from the LCM and TCGA cohorts (data not shown). However, none of the LCM cases carried the G84E SNP, and only five TCGA cases which carried the SNP (CH-5748, CH-5771, G9–6370, KK-A8IG, and V1-A9OF), were not amongst the MYC-high or MYC-low group (see Fig. 3c). Taken together, our data suggest that in MYC-high tumors, PCa development is collectively mediated by increased AR activity and *HOXB13* expression resulting from MEIS1 down-regulation.

## Discussion

In many cancer types, the role of amplified MYC in mediating tumorigenesis has been linked to genes involved in ribosomal biogenesis, universally up-regulated transcription, proliferation, and reprogramming cells to a pluripotent state [40]. A subset of advanced prostate cancers also harbor amplified MYC, but it is distinct from the up-regulated MYC that is a hallmark of many localized prostate cancers [3, 14–16]. In the current study, we used transcriptome profiling to assess subpopulations of prostate tumors based on differential MYC protein expression and MYC activity, and we similarly compared differentially expressed genes and pathways within the larger prostate TCGA cohort based on MYC activity. Finding that increased MYC activity was inversely proportional to overall transcription, we focused on down-regulated pathways, identifying a negative correlation between MYC activity and *MEIS1* expression, with MYC directly involved in the negative regulation of *MEIS1* as demonstrated by knockdown and chromatin immunoprecipitation analyses. The inverse association between *MEIS1* expression and AR extended further to *HOXB13* expression, indicating that in a subset of primary PCa, decreased expression of *MEIS1* may be necessary for AR and HOXB13 to drive tumor development and progression.

From its discovery as an oncogene to the subsequent challenges associated with targeting MYC pharmacologically, efforts have shifted in identifying targetable MYC effector genes or other targetable co-factors that are necessary for MYC activity [41, 42]. Efforts to dissect functions of MYC have frequently relied on cancer models in which *MYC* levels rise up to 20-fold, in contrast to 1–2 fold physiological elevation of *MYC* expression in PCa [2, 6, 43]. Not surprisingly, the phenotypes associated with *MYC* up-regulation differ. For example, *MYC* expression in the activation of lymphocytes [26] or in Burkitt’s lymphoma [27] is associated with a universal amplification of transcription, while we observed that in LNCaP PCa cells with up-regulated *MYC*, knocking down *MYC* by <50% with shRNA consistently increased the total amount of RNA produced per cell.

The differences in MYC function in PCa extend to the long-standing relationship between MYC and proliferation [40]. Here, we report a series of PCa tissues, serially-sectioned and stained with anti-MYC and anti-Ki67 antibodies, that show a weak proportional relationship. While up to 50% of luminal PCa cells were positive for MYC expression, proliferation measured by Ki67 affected <5% of cells. We show a similar relationship in the prostate TCGA cohort and PCa cell lines, suggesting that while proliferating cells may harbor MYC activity, MYC alone is not sufficient for proliferation in PCa. This is generally in agreement with findings that MYC expression alone is not reflective of an increased proliferative fraction of PCa cells [5].

A key finding from our study is the negative relationship between MYC activity and *MEIS1* expression. In the context of MYC’s role as a universal transcriptional amplifier, a target gene of MYC-mediated repression could simply be considered a technical anomaly reflecting unequal numbers of cells used for analysis [26]. However, we show that transcriptional signatures of MYC activity and *MEIS1* are inversely correlated in two large independent cohorts, which is largely consistent with the finding that tumors and PCa cells with increased *MEIS1* expression show decreased enrichment of MYC target gene sets by GSEA [19]. Importantly, we further demonstrate here with ChIP-seq that the effects on *MEIS1* expression are due in part to increased MYC occupancy at the *MEIS1* locus, such that by using shRNA to lower the level *MYC* with biological variability observed in patient samples, it resulted in repositioning of MYC at specific sites. However, the intensity of MYC binding at *MEIS1*, as measured by the peak height in IGV, is substantially less than the peak heights of known direct targets of MYC such as *PNP, NOLC1* and *EIF5A*, and the increased expression of *MEIS1* in the context of decreased *MYC* expression precludes *MYC* as a direct transcriptional activator. Indeed, *MEIS1* is a direct target of MIZ-1 [44], which functions to increase the expression of *MEIS1* in the absence of MYC. Therefore, one possible explanation for our observations is that a MYC-regulated transcriptional repressor also sits at the *MEIS1* promoter and competes with MYC for direct binding to MIZ-1 or another positive regulator of *MEIS1* transcription. In such a model, MYC would only be bound to *MEIS1* indirectly, and the interaction between MYC/MIZ-1 complexes and other transcriptional regulators of *MEIS1* is of interest for future study.

Previously, Bhanvadia et al. [19]. demonstrated that tumors with increased *MEIS1* are potentially less aggressive, based on studies of LAPC-4 PCa cells expressing shRNA against *MEIS1* and that tumors with lower levels of *MEIS1* were at greater risk of biochemical recurrence and metastasis. Specifically, they reported a step-wise decrease in *MEIS1* expression during PCa progression, but that knockdown of both *MEIS1* and *MEIS2* were necessary to suppress tumor development in vivo. HOXB13, a homeodomain transcription factor, has been shown to regulate AR activity while shRNA against *HOXB13* in LAPC4 cells inhibits their growth [18]. Based on these prior findings, as HOXB13 physically interacts with MEIS1 [18], tumors expressing less *MEIS1* would be expected to display greater *HOXB13* expression and AR activity. Indeed, we show an inverse relationship between *MEIS1* expression and AR activity in two independent cohorts, which is consistent with previous observations of a positive correlation between *MYC* expression and AR activity [7]. However, we did not observe a statistically significant inverse relationship between *MYC* and *MEIS2* (see Supplementary Table 2).

Our study has an important limitation in that we did not assess the relationship between *MYC* and *MEIS1* in additional human cell line or mouse model systems. Our focus on increased MYC activity in locoregional PCa is distinct from highly amplified *MYC* in metastatic PCa. The LNCaP model is the only commercially available PCa cell line that is genomically similar to localized PCa, derived from a regional lymph node, and is both androgen-dependent and luminal in origin. Mouse models with robustly overexpressed *MYC* and all other PCa cell lines derived from advanced metastases exhibit a high proliferative index, which does not accurately model early PCa, which is a slow-growing disease.

In summary, our analysis of MYC-expressing prostate tumors demonstrates an inverse relationship with *MEIS1* expression, which in turn is negatively correlated with *HOXB13* expression and AR activity. Mechanistically, our data demonstrate that *MEIS1* is a directly repressed target of MYC, and via effects on *HOXB13* link MYC activity to AR activity. The potential clinical significance of the inverse *MYC*/*MEIS1* relationship warrants further investigation as AR-directed therapies are introduced earlier in the clinical course of disease, and *MEIS1* levels may indicate potential sensitivity to treatment.

## Materials and methods

### Study approval

This research was conducted in accordance with the principles of the Declaration of Helsinki. All patients provided informed consent prior to participating in tissue procurement protocols, and all samples were deidentified as per institutional policies. The collection of radical prostatectomy specimens was approved by the Dana Farber/Harvard Cancer Center Institutional Review Boards, under protocol numbers 11–250, 15–008, and 15–492. The collection of prostate biopsy specimens was approved by the National Institutes of Health Institutional Review Board, under protocol number 15-c-0124. Tissues were fixed in formalin and embedded in paraffin according to standard procedures.

### Pan-cancer analysis

Gene-level normalized FPKM expression values for The Cancer Genome Atlas (TCGA) RNA-seq data were downloaded from the NCI Genomic Data Commons (https://gdc.cancer.gov). Cases were filtered for tumor samples within each organ type and cases with missing gene expression values were removed. Single-sample gene set enrichment analysis (ssGSEA) was performed on the GenePattern server (https://cloud.genepattern.org) using the ssGSEA Projection module version 9.1.1 with the following parameters: weighting exponent: 0.75; combine mode: combine.add; sample normalization method: none. For each TCGA tumor type, ssGSEA projection values were obtained for a 17-gene MYC-independent proliferation signature, a 54-gene MYC activity signature [22], and a prostate cancer-specific gene set generated by *MYC* overexpression in LNCaP cells [11]. The genes used for analysis are given in Supplementary Table 1.

### Cell lines and derivatives

RNA knockdown of *MYC* expression was achieved using the SMARTvector lentiviral shRNA system (Dharmacon) and a standard second-generation packaging system. hCMV-TurboGFP targeting vectors were MYC 512: V3SH11240-225190433 (GGTCGATGCACTCTGAGGC, targeting ORF); MYC 627: V3SH11240-226339523 (TTGATCATGCATTTGAAAC, targeting 3′ UTR); MYC 637: V3SH11240-226439183 (GTAGAAATACGGCTGCACC, targeting ORF); and non-targeting control: VSC11707. Briefly, 293FT cells (ThermoFisher) were reverse-transfected with a lentiviral shRNA expression vector in a 10 cm dish, using envelope (pCMV-VSV-G, #8454, Addgene) and packaging (pCMV-dR8.2 dvpr, #8455, Addgene) vectors following the ThermoFisher ViraPower protocols. Media containing lentivirus were harvested two days post-transfection, passed through a 0.45 μm filter, aliquoted and frozen. HT-1080 cells (ATCC) were used for titering virus following the ThermoFisher ViraPower protocol.

LNCaP cells (clone FGC, catalog number CRL1740) were purchased from ATCC. Cell line authentication by STR profiling and mycoplasma testing was performed every 6 months (Laragen, Inc). LNCaP cells were seeded in 6-well plates at 1 × 10^5^ cells per well and transduced with lentivirus at a multiplicity of infection of 1 in complete media for 24 h. Complete media were then replaced one day post-transduction, and selection with puromycin (1 μg/mL, Life Technologies) began 2 days post-transduction. Transduction was confirmed by visual confirmation of GFP fluorescence in >90% of cells, and knockdown was confirmed by qPCR and Western blotting.

### Statistical analysis

Statistical analyses were performed using GraphPad Prism 8 for Mac. Statistical tests used and relevant variables are indicated in the legend of each figure.

Additional methods including qPCR, western blotting, bioinformatic analyses, histology and ChIP-seq are provided in Supplementary Information.

## Supplementary information

Supplementary Information

Supplementary Table 1

Supplementary Table 2

Supplementary Table 3

Supplementary Table 4

Supplementary Table 5

Supplementary Table 6

## Data Availability

Human tissue RNA-seq data has been deposited into the Database of Genotypes and Phenotypes (https://dbgap.ncbi.nlm.nih.gov/), accession ID phs001813.v1.p1. Human tissue gene expression data has been deposited into the Gene Expression Omnibus (https://www.ncbi.nlm.nih.gov/geo/), accession ID GSE130046. ChIP-seq data has been deposited into the Sequence Read Archive (https://www.ncbi.nlm.nih.gov/sra), accession ID SRP218384 and GEO, accession ID GSE135942.

## Code availability

The code used to perform all analyses is available at https://github.com/CBIIT/lgcp.

## References

[1] Cancer Genome Atlas Research Network. (2015). The molecular taxonomy of primary prostate cancer. Cell.

[2] Taylor BS, Schultz N, Hieronymus H, Gopalan A, Xiao Y, Carver BS (2010). Integrative genomic profiling of human prostate cancer. Cancer Cell.

[3] Sowalsky AG, Kissick HT, Gerrin SJ, Schaefer RJ, Xia Z, Russo JW (2017). Gleason score 7 prostate cancers emerge through branched evolution of clonal gleason pattern 3 and 4. Clin Cancer Res.

[4] Trock BJ, Fedor H, Gurel B, Jenkins RB, Knudsen BS, Fine SW (2016). PTEN loss and chromosome 8 alterations in Gleason grade 3 prostate cancer cores predicts the presence of un-sampled grade 4 tumor: implications for active surveillance. Mod Pathol.

[5] Gurel B, Iwata T, M Koh C, Jenkins RB, Lan F, Van Dang C (2008). Nuclear MYC protein overexpression is an early alteration in human prostate carcinogenesis. Mod Pathol.

[6] Koh CM, Bieberich CJ, Dang CV, Nelson WG, Yegnasubramanian S, De Marzo AM (2010). MYC and prostate cancer. Genes Cancer.

[7] Bai S, Cao S, Jin L, Kobelski M, Schouest B, Wang X (2019). A positive role of c-Myc in regulating androgen receptor and its splice variants in prostate cancer. Oncogene.

[8] Packer JR, Maitland NJ (1863). The molecular and cellular origin of human prostate cancer. Biochimica et Biophysica Acta.

[9] Norris JD, Chang CY, Wittmann BM, Kunder RS, Cui H, Fan D (2009). The homeodomain protein HOXB13 regulates the cellular response to androgens. Mol Cell.

[10] Yang YA, Yu J (2015). Current perspectives on FOXA1 regulation of androgen receptor signaling and prostate cancer. Genes Dis.

[11] Barfeld SJ, Urbanucci A, Itkonen HM, Fazli L, Hicks JL, Thiede B (2017). c-Myc antagonises the transcriptional activity of the androgen receptor in prostate cancer affecting key gene networks. EBioMedicine.

[12] Barfeld SJ, Fazli L, Persson M, Marjavaara L, Urbanucci A, Kaukoniemi KM (2015). Myc-dependent purine biosynthesis affects nucleolar stress and therapy response in prostate cancer. Oncotarget.

[13] Koh CM, Gurel B, Sutcliffe S, Aryee MJ, Schultz D, Iwata T (2011). Alterations in nucleolar structure and gene expression programs in prostatic neoplasia are driven by the MYC oncogene. Am J Pathol.

[14] Baena-Del Valle JA, Zheng Q, Esopi DM, Rubenstein M, Hubbard GK, Moncaliano MC (2018). MYC drives overexpression of telomerase RNA (hTR/TERC) in prostate cancer. J Pathol.

[15] Robinson D, Van Allen Eliezer M, Wu Y-M, Schultz N, Lonigro Robert J, Mosquera J-M (2015). Integrative clinical genomics of advanced prostate cancer. Cell.

[16] Sowalsky AG, Ye H, Bhasin M, Van Allen EM, Loda M, Lis RT (2018). Neoadjuvant-intensive androgen deprivation therapy selects for prostate tumor foci with diverse subclonal oncogenic alterations. Cancer Res.

[17] Park JW, Lee JK, Phillips JW, Huang P, Cheng D, Huang J (2016). Prostate epithelial cell of origin determines cancer differentiation state in an organoid transformation assay. Proc Natl Acad Sci USA.

[18] Johng D, Torga G, Ewing CM, Jin K, Norris JD, McDonnell DP (2019). HOXB13 interaction with MEIS1 modifies proliferation and gene expression in prostate cancer. Prostate.

[19] Bhanvadia RR, VanOpstall C, Brechka H, Barashi NS, Gillard M, McAuley EM (2018). MEIS1 and MEIS2 expression and prostate cancer progression: a role for HOXB13 binding partners in metastatic disease. Clin Cancer Res.

[20] Brechka H, Bhanvadia RR, VanOpstall C, Vander Griend DJ (2017). HOXB13 mutations and binding partners in prostate development and cancer: function, clinical significance, and future directions. Genes Dis.

[21] Chen H, Liu H, Qing G (2018). Targeting oncogenic Myc as a strategy for cancer treatment. Signal Transduct Target Ther.

[22] Shaffer AL, Wright G, Yang L, Powell J, Ngo V, Lamy L (2006). A library of gene expression signatures to illuminate normal and pathological lymphoid biology. Immunological Rev.

[23] Hammarsten P, Josefsson A, Thysell E, Lundholm M, Hagglof C, Iglesias-Gato D (2019). Immunoreactivity for prostate specific antigen and Ki67 differentiates subgroups of prostate cancer related to outcome. Mod Pathol.

[24] Tretiakova MS, Wei W, Boyer HD, Newcomb LF, Hawley S, Auman H (2016). Prognostic value of Ki67 in localized prostate carcinoma: a multi-institutional study of >1000 prostatectomies. Prostate Cancer Prostatic Dis.

[25] Cunningham D, You Z (2015). In vitro and in vivo model systems used in prostate cancer research. J Biol Methods.

[26] Nie Z, Hu G, Wei G, Cui K, Yamane A, Resch W (2012). c-Myc is a universal amplifier of expressed genes in lymphocytes and embryonic stem cells. Cell.

[27] Lin CY, Lovén J, Rahl PB, Paranal RM, Burge CB, Bradner JE (2012). Transcriptional amplification in tumor cells with elevated c-Myc. Cell.

[28] Junttila MR, Westermarck J (2008). Mechanisms of MYC stabilization in human malignancies. Cell Cycle.

[29] Hawksworth D, Ravindranath L, Chen Y, Furusato B, Sesterhenn IA, McLeod DG (2010). Overexpression of C-MYC oncogene in prostate cancer predicts biochemical recurrence. Prostate Cancer Prostatic Dis.

[30] Marcos S, González-Lázaro M, Beccari L, Carramolino L, Martin-Bermejo MJ, Amarie O (2015). Meis1 coordinates a network of genes implicated in eye development and microphthalmia. Development.

[31] Wang GG, Pasillas MP, Kamps MP (2005). Meis1 programs transcription of FLT3 and cancer stem cell character, using a mechanism that requires interaction with Pbx and a novel function of the Meis1 C-terminus. Blood.

[32] Calvo KR, Sykes DB, Pasillas MP, Kamps MP (2002). Nup98-HoxA9 immortalizes myeloid progenitors, enforces expression of Hoxa9, Hoxa7 and Meis1, and alters cytokine-specific responses in a manner similar to that induced by retroviral co-expression of Hoxa9 and Meis1. Oncogene.

[33] Takeda A, Goolsby C, Yaseen NR (2006). NUP98-HOXA9 induces long-term proliferation and blocks differentiation of primary human CD34+ hematopoietic cells. Cancer Res.

[34] Collins CT, Hess JL (2016). Deregulation of the HOXA9/MEIS1 axis in acute leukemia. Curr Opin Hematol.

[35] Yohe ME, Gryder BE, Shern JF, Song YK, Chou H-C, Sindiri S (2018). MEK inhibition induces MYOG and remodels super-enhancers in RAS-driven rhabdomyosarcoma. Sci Transl Med.

[36] Gallenne T, Ross KN, Visser NLS, Desmet CJ, Wittner BS (2017). Systematic functional perturbations uncover a prognostic genetic network driving human breast cancer. Oncotarget.

[37] Dunham I, Kundaje A, Aldred SF, Collins PJ, Davis CA, Doyle F (2012). An integrated encyclopedia of DNA elements in the human genome. Nature.

[38] Cui L, Li M, Feng F, Yang Y, Hang X, Cui J (2014). MEIS1 functions as a potential AR negative regulator. Exp Cell Res.

[39] Mendiratta P, Mostaghel E, Guinney J, Tewari AK, Porrello A, Barry WT (2009). Genomic strategy for targeting therapy in castration-resistant prostate cancer. J Clin Oncol.

[40] Dang CV (2012). MYC on the path to cancer. Cell.

[41] Dang CV (1999). c-Myc target genes involved in cell growth, apoptosis, and metabolism. Mol Cell Biol.

[42] Delmore JE, Issa GC, Lemieux ME, Rahl PB, Shi J, Jacobs HM (2011). BET bromodomain inhibition as a therapeutic strategy to target c-Myc. Cell.

[43] Neiman PE, Ruddell A, Jasoni C, Loring G, Thomas SJ, Brandvold KA (2001). Analysis of gene expression during myc oncogene-induced lymphomagenesis in the bursa of Fabricius. Proc Natl Acad Sci USA.

[44] Varlakhanova N, Cotterman R, Bradnam K, Korf I, Knoepfler PS (2011). Myc and Miz-1 have coordinate genomic functions including targeting Hox genes in human embryonic stem cells. Epigenetics Chromatin.

