# Supplementary material for: MEIS1 down-regulation by MYC mediates prostate cancer development through elevated HOXB13 expression and AR activity
Source: Oncogene. 2020 Jul 17;39(34):5663–74. doi: 10.1038/s41388-020-01389-7 (PMC7441006; doi:10.1038/s41388-020-01389-7)
Supplement: Supplementary file 1 — Supplementary Information [file 41388_2020_1389_MOESM1_ESM.pdf]

# *MEIS1* down-regulation by MYC mediates prostate cancer development through elevated *HOXB13* expression and AR activity

Nichelle C. Whitlock[1], Shana Y. Trostel[1], Scott Wilkinson[1], Nicholas T. Terrigino[1], S. Thomas Hennigan[1], Ross Lake[1], Nicole V. Carrabba[1], Rayann Atway[1], Elizabeth D. Walton[1], Berkley E. Gryder[2], Brian J. Capaldo[1], Huihui Ye[3], *Adam G. Sowalsky[1]

[1]Laboratory of Genitourinary Cancer Pathogenesis, National Cancer Institute, NIH, Bethesda, MD, 20892
[2]Genetics Branch, National Cancer Institute, NIH, Bethesda, MD, 20892
[3]Department of Pathology, Beth Israel Deaconess Medical Center, Boston, MA, 02215

## Supplementary Information

Supplementary Materials and Methods
Supplementary Tables
Supplementary Figures
Supplementary References

## Supplementary Materials and Methods

*Quantitative polymerase chain reaction*

RNA was purified using the RNeasy Plus Mini Kit (Qiagen) and quality tested using the High Sensitivity RNA ScreenTape (TapeStation 4200, Agilent Technologies). Expression levels of *MYC* and *MEIS1* transcript were quantified using TaqMan Fast Virus 1-Step Master Mix (Life Technologies) using PrimeTime qPCR Probe Assays from Integrated DNA Technologies in a 3:1 ratio of primer to 5′ 6-FAM and 3′ TAMRA labeled probe. Sequences of the primer-probe sets are listed in Supplementary Table 7. qPCR was performed in duplex with GAPDH endogenous control TaqMan assays (Life Technologies), and relative transcript levels were quantified using the $2^{-\Delta\Delta CT}$ method [1].

*Western blotting*

Total cell lysates were isolated using RIPA buffer (Pierce) containing protease and phosphatase inhibitors (Pierce). 15 μg of soluble protein per well were separated by SDS-PAGE and transferred to nitrocellulose membranes. Blots were blocked for 1 hour in 5% nonfat dry milk in TBS/0.05% Tween-20 (TBS-T) and incubated with primary antibodies at 4°C overnight. Primary antibodies used were anti-MYC (Y69; ab32072, Abcam, 1:2000 dilution) and anti-GAPDH (6C5; MAB374, Millipore Sigma, 1:1000 dilution). After washing with TBS-T, the blots were incubated with anti-rabbit or anti-mouse horseradish peroxidase-conjugated secondary antibody (Jackson ImmunoResearch, 1:10,000 dilution) for 1 hour and washed 4 times. Chemiluminescence was detected with Western Lightning Plus ECL (Perkin Elmer) and recorded using a ChemiDoc Imaging System (Bio-Rad).

*Gene expression analysis*

Whole transcriptome profiling of LCM tissue was performed at the CCR Illumina Sequencing Facility using the Illumina HiSeq v4 sequencing system to a depth of 50 million reads (50 cycles, paired-end). Sample reads were trimmed for adapters using Trim Galore before alignment to the GRCh37 reference genome using STAR [2] with default parameters. Aligned reads were quantified using featureCounts [3] with default parameters, and differential expression analysis between MYC-high and MYC-low samples was performed using edgeR [4]. The $\log_2$ counts per million (CPM) values from the output were used for downstream analysis.

From the TCGA PRAD dataset used in the pan-cancer analysis, the 20 cases with highest MYC activity signature and the 20 cases with the lowest MYC activity signature were re-analyzed using the same pipeline as tissue for determining differentially expressed genes. FASTQ files for those cases were retrieved from the NCI Genomic Data Commons via access to dbGaP phs000178.

Gene set enrichment analysis (GSEA) was performed in clusterProfiler [5] comparing MYC-high versus MYC-low LCM foci and TCGA cases, and run against gene sets in the Molecular Signature Database (v6.2). The AR activity signature gene set is given in Supplementary Table 1.

*Immunohistochemistry (IHC)*

Serial sections of formalin-fixed, paraffin-embedded tissues were cut at 6 μm thickness onto Superfrost Plus slides (Fisher Scientific). Slides were stained with the following: hematoxylin & eosin (H&E); anti-ERG (EPR3864; Abcam Cat# ab92513, 1:100 dilution), anti-MYC (Y69; Abcam Cat# ab32072, 1:100 dilution); anti-Ki67 (D2H10; Cell Signaling Cat# 9027, 1:100 dilution), anti-PTEN (D4.3; Cell Signaling Cat#9188, 1:100 dilution); and PIN-4 cocktail (ready-to-use; Biocare Medical Cat# PPM225DS). Antigen retrieval was performed using a pre-heated steamer for 20 minutes in Diva Decloaker (Biocare Medical, Cat# DV2004MX) for Ki67, ERG, PTEN and PIN-4 or High pH Antigen Retrieving Solution (Abcam, Cat# 972) for MYC, followed by 20 minutes of cooling. Sections were blocked with hydrogen peroxide (Sigma-Aldrich, Cat# 216763) for 5

minutes, blocked with Background Sniper (Biocare Medical, Cat# BS966) for 10 minutes for PIN-4 or VectaStain Elite ABC HRP kit (Vector Laboratories, Cat# PK-6101) for MYC, Ki67, PTEN and ERG, and incubated with primary antibody overnight at 4°C (MYC, Ki67, ERG and PTEN) or for one hour (PIN-4). Secondary labeling was performed using Mach 2 Double Stain for PIN-4 (Biocare Medical, Cat# MRCT525) or the VectaStain Elite ABC HRP kit for MYC, Ki67, ERG and PTEN for 30 minutes. Avidin-biotin complexing was then performed for 30 minutes for MYC, Ki67, ERG and PTEN. Colorimetric detection was achieved using DAB Peroxidase HRP (Vector Laboratories, Cat# SK4100) for MYC, Ki67, ERG and PTEN, Betazoid DAB (Biocare Medical, Cat# BDB2004) for PIN-4, and Vulcan Red Fast Chromogen (Biocare Medical, Cat# FR805) for PIN-4. Counterstaining was performed using Mayer's Hematoxylin Solution (Sigma Aldrich, Cat# MHS16). PIN-4 stained slides were air-dried. MYC, Ki67, ERG and PTEN stained slides were dehydrated through graded alcohol and cleared in xylenes. Slides were mounted using Permount (ThermoFisher).

*Laser capture microdissection and RNA purification*

Laser capture microdissection (LCM) was performed as previously described [6]. For within-patient analyses (high and low MYC levels in the same patient), cases were selected based on having regions of differential MYC staining intensity by IHC, but concordant ERG and PTEN status by IHC. Areas of high MYC were defined by moderate (++) or high (+++) staining in at least 30% of cancer cells, while areas of low MYC were defined by weak (+) or absent (-) staining in at least 75% of cancer cells, with intense staining limited to 10% of cancer cells. LCM was guided by review of serially-sectioned slides stained with MYC, visualized using ZEN Browser (Zeiss) on an adjacent monitor as reference. High or low MYC cancer glands from each case were collected on separate caps and a photomicrograph was acquired for the purposes of estimating tumor cell purity in each sample. For between-patient analyses, MYC status was ascertained by IHC and assigned to the foci of tumor cells subjected to LCM. The distinction between within-patient and between-patient analyses is that all within-patient cases harbored both MYC-high and MYC-low populations of tumor cells.

RNA was extracted using the RNeasy FFPE Kit (Qiagen) following the manufacturer's protocol with modifications. Briefly, the film to which LCM cells adhered was removed using a disposable blade, immersed into Buffer PKD with proteinase K, digested for 15 min at 56°C, followed by a 15-minute incubation at 80°C. After centrifugation and DNase treatment to remove genomic DNA, concentrated RNA was purified using RNeasy MinElute spin columns and eluted with 30 μL RNase-free water. RNA yields were quantified using RiboGreen reagent (Life Technologies).

*Computer-aided image analysis*

Stained slides were scanned using the 20× objective (Plan-Apochromat, NA 0.8) with brightfield illumination on an AxioScan.Z1 (Zeiss). All slides were reviewed by a board-certified surgical pathologist following the 2014 ISUP guidelines [7]. Whole slide .CZI files of Ki67- and MYC-stained slides were imported into Definiens Developer XD 64. The magnification for each analysis was set to 40×, with 0.11 μm/pixel for both Ki67 and MYC solutions. IHC stain was identified as brown chromogen. The tumor cells were counted using their nuclear stain.

For Ki67, composer magnification was set to 6× with 12 training subsets at segmentation level 9. Segments were classified as tumor or stroma, with normal glands excluded. Within the tumor pattern, cellular analysis magnification was set to 10× with 12 training subsets, and within nuclear detection, thresholds were 0.2 for hematoxylin and 0.5 for brown chromogen with typical nuclear size set to 30 μm. Nucleus classification: low vs. medium at 0.65; medium vs. high at 0.86.

For MYC analysis, composer magnification was set to 7.5× with 12 training subsets at segmentation level 7. Segments were classified as tumor or stroma, with normal glands excluded. Within the tumor pattern, cellular analysis magnification was set to 10× with 12 training subsets, and

within nuclear detection, thresholds were 0.1 for hematoxylin and 0.45 for brown chromogen: 0.45 with typical nuclear size set to 30 μm. Nucleus classification: low vs. medium at 0.5; medium vs. high at 0.7.

The total number of positively stained nuclei were reported, along with distribution of low-, medium-, and high-intensity stained nuclei for each tumor focus. A percent positive index score was calculated using a weighted average divided by the total number of nuclei, where *index* = [ (1 × nuclei stained low) + (2 × nuclei stained medium) + (3 × nuclei stained high) ] ÷ (3 × total nuclei).

*ChIP-seq analysis*

ChIP was performed according to the ChIP-IT High Sensitivity Kit (Active Motif) protocol. Briefly, cultured LNCaP cells with control or MYC knockdown constructs were crosslinked, quenched, lysed, and the chromatin sheared to a size between 200-1000 bp. *Drosophila melanogaster* chromatin and antibody spike-in controls were added per the protocol. For each ChIP, 30 μg of chromatin were incubated with anti-MYC antibody (ab32072, Abcam, 1:100 dilution) on an end-to-end rotator overnight at 4°C. Antibody-bound protein/chromatin complexes were immunoprecipitated with Protein A/G beads, reverse-crosslinked, and DNA purified. Quality of ChIP-enriched DNA was assessed using the ChIP-IT qPCR Analysis Kit (Active Motif) per the manufacturer's protocol, using primers for the MYC super-enhancer. Primer sequences are given in Supplementary Table 7.

Purified ChIP DNA was assembled into libraries using the TruSeq ChIP Library Preparation Kit (Illumina) according to the manufacturer's protocol, pooled in equimolar ratios, and sequenced on a NextSeq 500 (Illumina) with 76 cycles of single-end sequencing. All samples had 90% of bases at Q30 or above, with yields between 26-42 million pass filter reads per sample. Samples were adaptor-trimmed with Trim Galore (https://github.com/FelixKrueger/TrimGalore) and reads were mapped to the human genome GRCh37 and *Drosophila melanogaster* genome dm6 with BWA-MEM [8]. Aligned reads were deduplicated using Picard Tools (https://broadinstitute.github.io/picard). Peak calling was performed using MACS2 [9] callpeak with the parameters -g hs and -q 0.01, differential binding of peaks was ascertained using DiffBind [10] with default parameters, and motif analysis was performed using HOMER [11] findMotifsGenome.pl with the parameters -size 200 and -len 8,10,12. To normalize for technical and genome-wide variability, reads mapping to dm6 were used to calculate reads per by million mapped dm6 reads (RRPM) as described [12].

*Germline SNP analysis*

Buffy coat or saliva were acquired from patients in the LCM cohort. DNA was extracted using the Qiagen QIAamp blood mini kit or the GenoTek Oragene DNA extraction kit. Genomic DNA was then sheared and assembled into paired-end Illumina-compatible libraries using either the NEBNext library preparation kit (for whole-genome sequencing) or Agilent SureSelect Human All Exon V5 kit (for whole-exome sequencing). Pass filter FASTQ files were adaptor-trimmed. TCGA PRAD BAM files (with the normal tissue barcode) were downloaded from the NCI Genomic Data Commons and processed with PICARD SamToFastq.

FASTQ files from both cohorts were aligned to the b37+decoy version of the human genome with BWA-MEM 0.7.17 and processed through the SPARK implementation of GATK 4.1.3.0, which includes the modules MarkDuplicatesSpark, PICARD SetNmMdAndUqTags, and BQSRPipelineSpark. Germline VCF files were generated by running HaplotypeCaller on each BAM file individually, and then cohort calling using CombineGVCFs, GenotypeGVCFs, VariantRecalibrator and ApplyVQSR to indel and SNV training sets. Annotation of VCF files with Oncotator revealed the presence or absence of the *HOXB13* G84E mutation in each sample.

**Supplementary Tables**

**Supplementary Table 1**
See Microsoft Excel file.

**Supplementary Table 1.** Genes used for the 17-gene proliferation signature, 54-gene MYC activity signature, 607-gene prostate cancer-specific MYC overexpression gene set, and 266-gene AR activity signature.

**Supplementary Table 2**
See Microsoft Excel file.

**Supplementary Table 2.** Differentially expressed genes from laser capture microdissected tissues comparing MYC-high versus MYC-low protein expression by immunohistochemistry are ranked by average $\log_2$ fold-change values, shown with a $-\log_{10}$ false-discovery rate that was calculated using the Benjamini-Hochberg correction of a quasi-likelihood framework F test with edgeR.

**Supplementary Table 3**
See Microsoft Excel file.

**Supplementary Table 3.** Differentially expressed genes from the prostate TCGA comparing MYC-high versus MYC-low activity by gene expression signature scoring are ranked by average $\log_2$ fold-change values, shown with a $-\log_{10}$ false-discovery rate that was calculated using the Benjamini-Hochberg correction of a quasi-likelihood framework F test with edgeR.

**Supplementary Table 4**
See Microsoft Excel file.

**Supplementary Table 4.** Overlap of differentially-expressed genes from Supplementary Tables 3 and 4. Statistical significance is based on a false-discovery rate cutoff of 0.1 for tissue and 0.05 for TCGA.

**Supplementary Table 5**
See Microsoft Excel file.

**Supplementary Table 5.** Overlap of significantly enriched mSigDB gene sets based on gene set enrichment analysis of MYC high versus MYC low tissue and TCGA $\log_2$ fold-change gene expression values, containing at least one differentially expressed gene from Supplementary Tables 3 and 4. Data shown reflect an enrichment *Q* value cut-off of 0.05.

**Supplementary Table 6**
See Microsoft Excel file.

**Supplementary Table 6**. Peak overlap between MYC ChIP-seq experiments and publically-available MYC ChIP-seq datasets.

**Supplementary Table 7**

| Gene | Primer/Probe | Sequence |
|---|---|---|
| *MYC* (exon 1-2) | | |
| | Primer 1 | 5′-CAGTAGAAATACGGCTGCAC-3′ |
| | Primer 2 | 5′-TTCGGGTAGTGGAAAACCAG-3′ |
| | Probe | 5′-6-FAM-CCGCGACGATGCCCCTCAA-TAM-3′ |
| *MYC* (exon 2-3) | | |
| | Primer 1 | 5′-TCTTCCTCATCTTCTTGTTCCTC-3′ |
| | Primer 2 | 5′-TCCTCGGATTCTCTGCTCTC-3′ |
| | Probe | 5′-6-FAM-TGGGCGGTGTCTCCTCATGG-TAM-3′ |
| *MEIS1* (exon 6-7) | | |
| | Primer 1 | 5′-TGGTGATAGACGATAGAGAAGGA-3′ |
| | Primer 2 | 5′-ATGCCGTGTCATCATGATCTC-3′ |
| | Probe | 5′-6-FAM-CCAAGAGGGCTGGTCAGTTAGATTTGC-TAM-3′ |
| *MEIS1* (exon 10-12) | | |
| | Primer 1 | 5′-AGAATAGTGCAGCCCATGATAG-3′ |
| | Primer 2 | 5′-TTCCACTCATAGGTCCTGGT-3′ |
| | Probe | 5′-6-FAM-CCTTGACTTACTGCTCGGTTGGACT-TAM-3′ |
| *MYC SE* (ChIP qPCR) | | |
| | Forward | 5′-CCACCCTCCTCCGATCTAG-3′ |
| | Reverse | 5′-CCCAGCAGTGCTACATGATC-3′ |

**Supplementary Table 7.** Sequences of primer-probe or primer sets used for qPCR.

# Supplementary Figures

## Supplementary Figure 1

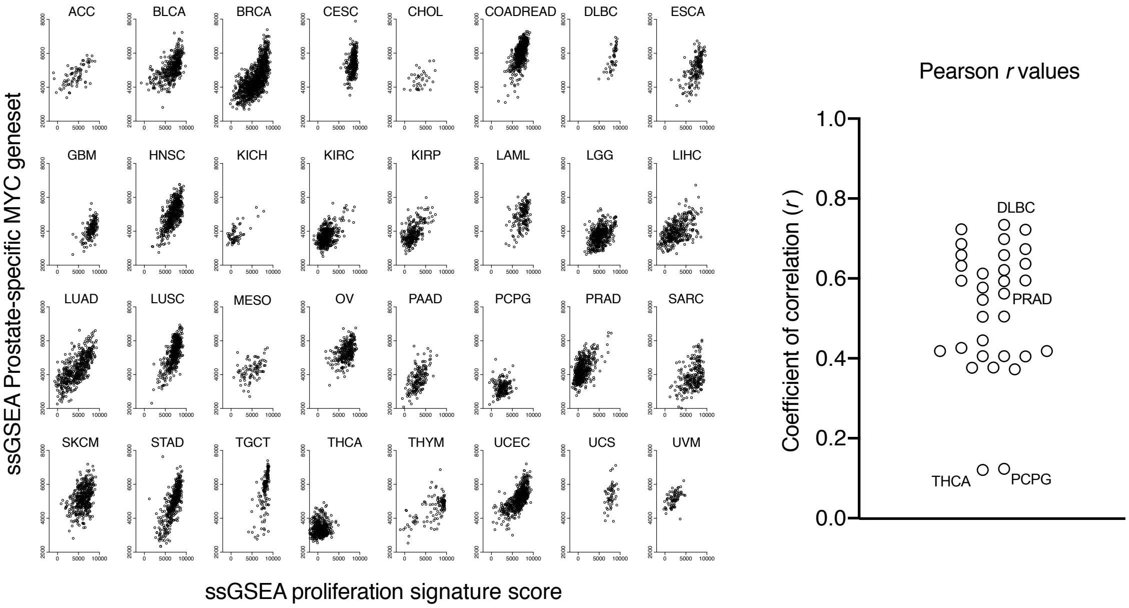


*Supplementary Figure 1. MYC and proliferation in human cancers.*

A. Left: correlation of a 607-gene prostate cancer-specific MYC overexpression ssGSEA score and the 17-gene ssGSEA proliferation signature score for each tumor type from the TCGA pan-cancer cohort. All scatter plots are on the same scale. Right: plot of Pearson *r* coefficients of correlation from each scatter plot shown on the left. All tumor types showed a positive correlation, ranging from 0.127 (THCA: thyroid carcinoma) to 0.7325 (DLBC: diffuse large B-cell lymphoma). PRAD: prostate adenocarcinoma, $r = 0.5609$. All correlations were significant at $P < 0.0001$ except CHOL ($P = 0.0238$), PCPG ($P = 0.0998$), THCA ($P = 0.0070$) and UCS ($P = 0.0039$).

**Supplementary Figure 2**

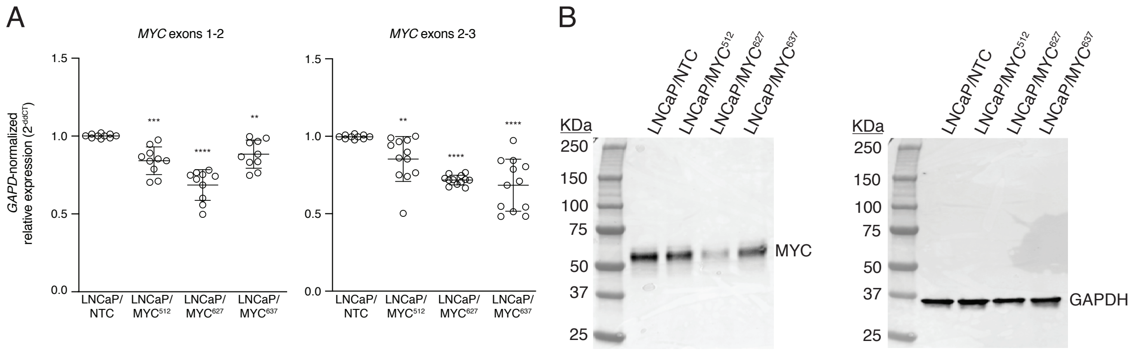


*Supplementary Figure 2. Knockdown of MYC in LNCaP cells with shRNA.*

LNCaP cells were transduced with the indicated viral particles expressing non-targeting (NTC) or MYC-knockdown hairpins. After two weeks of selection, lysates were harvested. A. Quantitative reverse-transcription PCR of *GAPD*-normalized *MYC* transcript in LNCaP *MYC* knockdown cell lines relative to control, measuring across the splice boundary of exons 1-2 (left) or exons 2-3 (right). Bars and whiskers represent the mean ± standard deviation of ten independent experiments conducted in triplicate, plotted individually as open circles (**, $P < 0.01$; ***, $P < 0.001$; ****, $P < 0.0001$ by Student's *t* test). B. Representative uncropped immunoblots of MYC knockdown LNCaP cell lysates with anti-MYC and anti-GAPDH. Protein molecular weight is given by marker sizes on the left.

**Supplementary Figure 3**

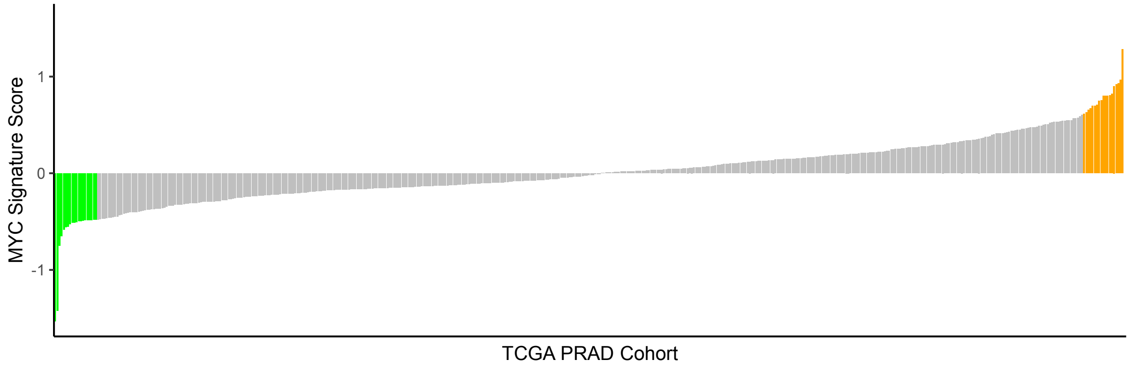


*Supplementary Figure 3. Depiction of TCGA cases with high or low MYC activity.*

All prostate TCGA cases were evaluated with an average median absolute deviation-modified *z*-score to determine individual sample MYC activity scores from mRNA expression data. Cases are ranked low to high, with the lowest scoring 20 samples in green and the highest scoring 20 samples in orange.

**Supplementary Figure 4**

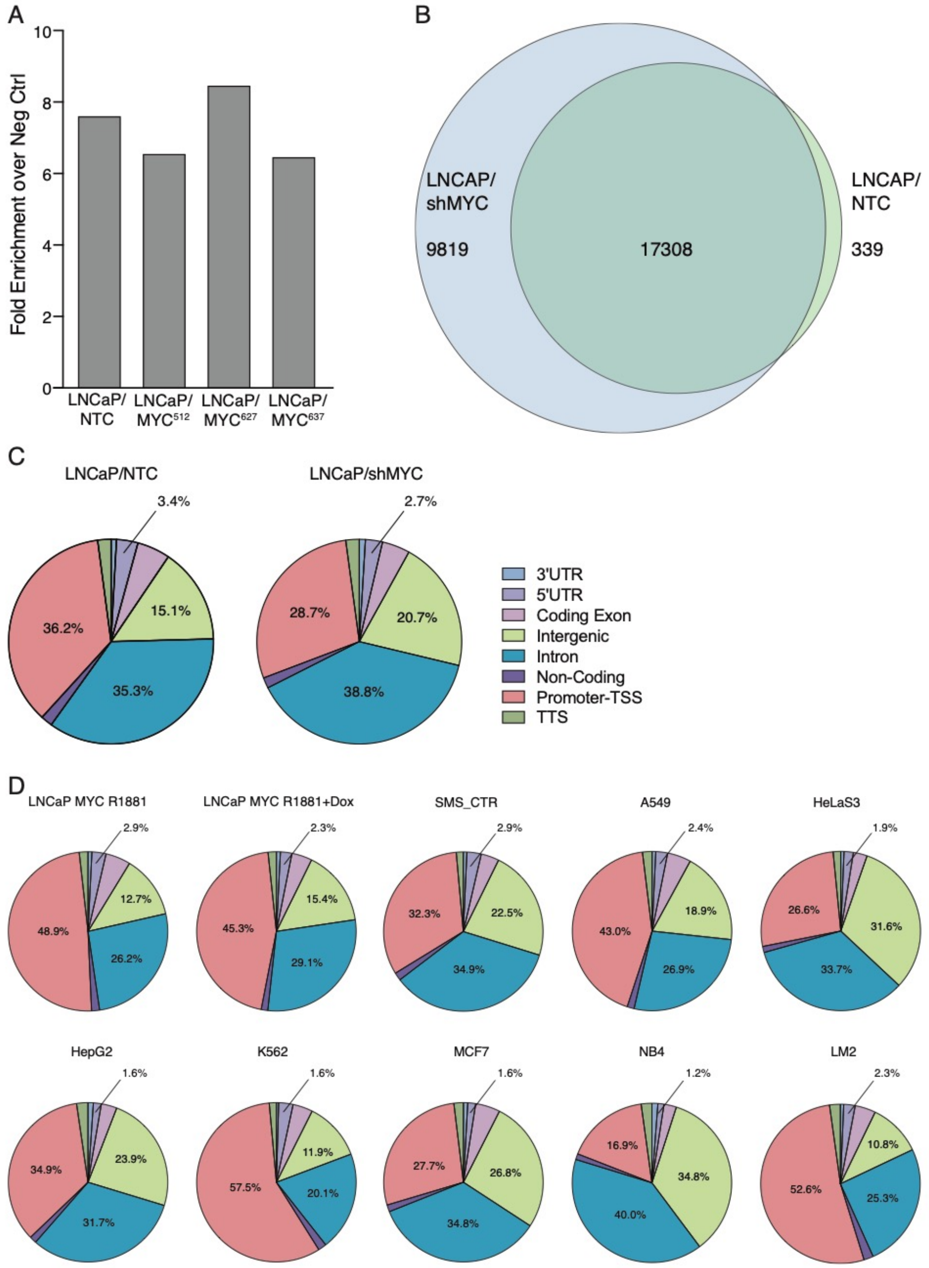


*Supplementary Figure 4. Technical and functional validation of MYC ChIP-seq of LNCaP/NTC and LNCaP/shMYC cells.*

A. Primers amplifying a portion of the *MYC* super enhancer were used to verify enrichment of known MYC binding sites following ChIP prior to ChIP-seq library assembly. Fold enrichment was determined by dividing the MYC signal by the signal from negative control primers. B. Venn diagram depicting the overlaps between LNCaP/NTC and LNCaP/shMYC MYC ChIP-seq datasets. C. MYC-bound sites from LNCaP/NTC and all three combined LNCaP/shMYC ChIP-seq experiments were annotated and plotted as a percentage of all peaks. D. MYC-bound sites from publicly-available MYC ChIP-seq studies were annotated and plotted as a percentage of all peaks. Datasets used for analysis were downloaded from the NCBI GEO data repository: GSE31477, GSE33213 (Dunham et al., 2012), GSE73994 (Barfeld et al., 2017), GSE85169 (Yohe et al., 2018), and GSE95303 (Gallenne et al., 2017).

**Supplementary Figure 5**

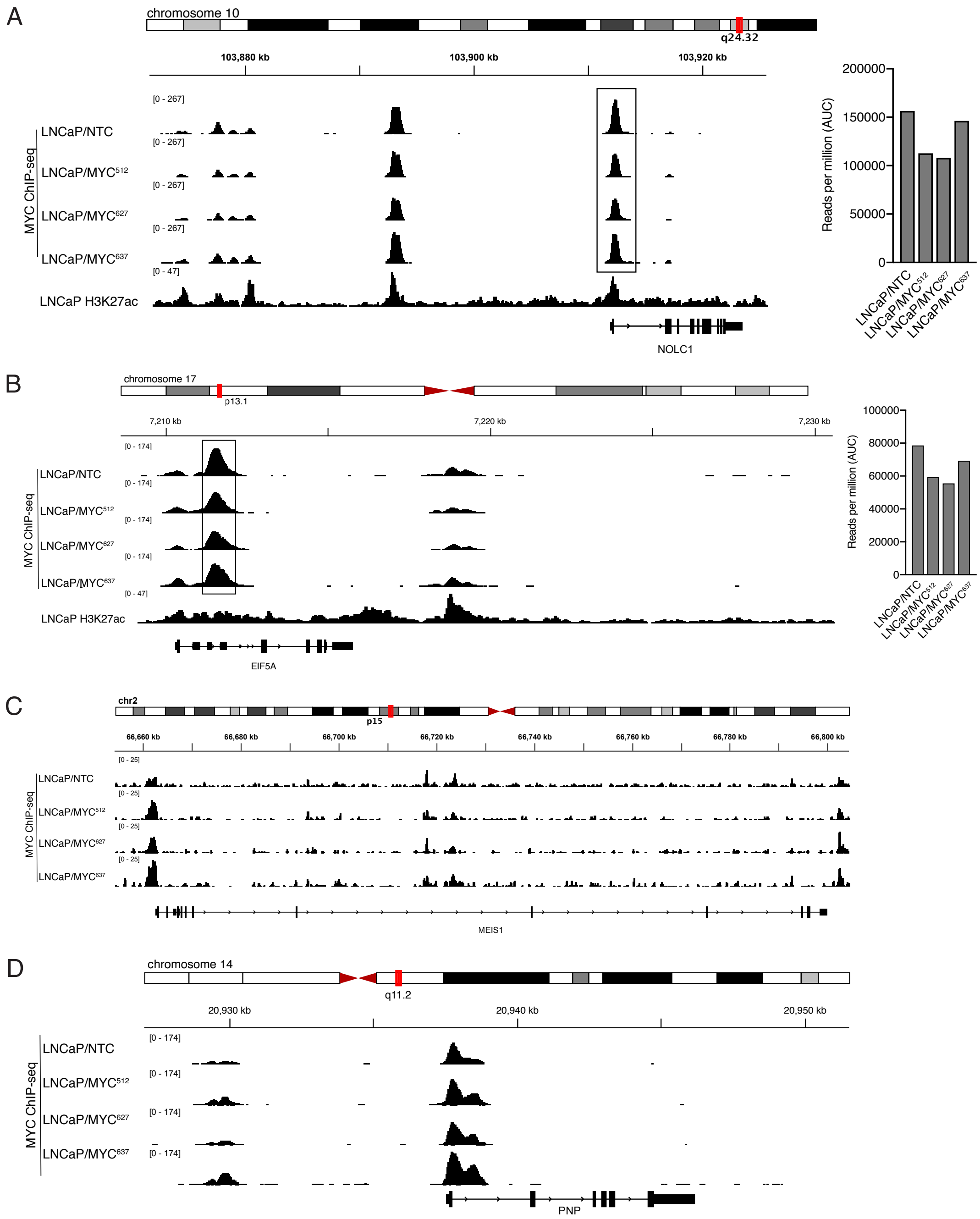


*Supplementary Figure 5. Effects of decreased MYC expression on MYC binding at target gene loci.*

A. IGV depiction of MYC and H3K27ac binding events in LNCaP *MYC* knockdown cell lines, showing decreased MYC occupancy at the *NOLC1* locus in cells harboring *MYC* knockdown. B. IGV depiction of MYC and H3K27ac binding events in LNCaP *MYC* knockdown cell lines, showing decreased MYC occupancy at the *EIF5A* locus in cells harboring *MYC* knockdown. Graphs to the right of (A) and (B) depict the area under the curve (AUC) of read counts for the MYC peaks outlined by the boxes. C. IGV depiction of binding events in LNCaP *MYC* knockdown cell lines at the *MEIS1* locus when normalized to reads mapping to *Drosophila* spike-in chromatin. D. IGV depiction of binding events in LNCaP *MYC* knockdown cell lines at the *PNP* locus when normalized to reads mapping to *Drosophila* spike-in chromatin.

**Supplementary References**

1 Livak KJ, Schmittgen TD. Analysis of Relative Gene Expression Data Using Real-Time Quantitative PCR and the 2−ΔΔCT Method. *Methods* 2001; 25: 402-408.

2 Dobin A, Davis CA, Schlesinger F, Drenkow J, Zaleski C, Jha S *et al*. STAR: ultrafast universal RNA-seq aligner. *Bioinformatics* 2013; 29: 15-21.

3 Liao Y, Smyth GK, Shi W. featureCounts: an efficient general purpose program for assigning sequence reads to genomic features. *Bioinformatics* 2013; 30: 923-930.

4 Robinson MD, McCarthy DJ, Smyth GK. edgeR: a Bioconductor package for differential expression analysis of digital gene expression data. *Bioinformatics* 2009; 26: 139-140.

5 Yu G, Wang L-G, Han Y, He Q-Y. clusterProfiler: an R Package for Comparing Biological Themes Among Gene Clusters. *OMICS: A Journal of Integrative Biology* 2012; 16: 284-287.

6 Sowalsky AG, Ye H, Bubley GJ, Balk SP. Clonal Progression of Prostate Cancers from Gleason Grade 3 to Grade 4. *Cancer Research* 2013; 73: 1050.

7 Epstein JI, Egevad L, Amin MB, Delahunt B, Srigley JR, Humphrey PA. The 2014 International Society of Urological Pathology (ISUP) Consensus Conference on Gleason Grading of Prostatic Carcinoma. *The American Journal of Surgical Pathology* 2016; 40: 244-252.

8 Li H. Aligning sequence reads, clone sequences and assembly contigs with BWA-MEM. *arXiv preprint* 2013.

9 Zhang Y, Liu T, Meyer CA, Eeckhoute J, Johnson DS, Bernstein BE *et al*. Model-based Analysis of ChIP-Seq (MACS). *Genome Biology* 2008; 9: R137.

10 Stark R, Brown GD. DiffBind: differential binding analysis of ChIP-seq peak data. *Bioconductor* 2011.

11 Heinz S, Benner C, Spann N, Bertolino E, Lin YC, Laslo P *et al*. Simple combinations of lineage-determining transcription factors prime cis-regulatory elements required for macrophage and B cell identities. *Mol Cell* 2010; 38: 576-589.

12 Hennigan ST, Trostel SY, Terrigino NT, Voznesensky OS, Schaefer RJ, Whitlock NC *et al*. Low Abundance of Circulating Tumor DNA in Localized Prostate Cancer. *JCO Precision Oncology* 2019; 3: 1-13.
